# Monthly Summary

**Published:** 1870-03

**Authors:** 


### MONTHLY SUMMARY.

*The Perils of Fashion.*—We are indebted to a lady correspondent for the correction of a mistake, or rather an omission, in our recent article on Tight-lacing. In ascribing the ungainly, feeble, and tottering walk of our modern fine ladies and their middle-class imitators to the decrepitude induced by tight-lacing, we omitted to mention another fashionable folly which assists in the production of this evil, and has also other sins of its own to answer for. The custom of wearing high boot-heels, and those too so much smaller than the actual heel of the wearer as to afford no solid support, but only a balancing-point, is a source of much mischief. In the first place, it throws the centre of gravity of the body so far forward that a free and gracefully erect carriage is impossible. Secondly, there being no firm support to the heel, ladies are very apt to twist the ankle suddenly by overbalancing themselves; and this is not only bad in itself, but the fear of its ocurrrence makes them assume a timid, mincing gait. And thirdly, the effect of driving the foot constantly forward into toe of the boot is to produce a very ugly and painful distortion to the great toe joint.

There is little need for wonder at the almost fierce contempt with which young men whose characters are at all above the lowest grades of conventional inanity regard the average "girl of the period." It cannot be denied that there is a significant correspondence between the æsthetic hideousness and the degrading effects on physical health which are produced by tight stays and crippling boots, and a certain mental and moral tone in female society of the present day, which is no less surprising than it is repulsive. The whole dress and carriage of our fashionable women, for several years past, has been modelling itself, with less and less concealment, upon the ideal furnished by Parisian *lorettes* of the consumptive Traviata type. It is not our business to set up as moral censors. But we may be excused if, for once in a way, we find it impossible to ignore the logical though repulsive consistency of the *grandes dames* and citizenesses who are willing to spoil their lungs and their digestions, and endanger their chances of happy maternity, for the sake of a wasp waist; to talk slang closely verging on indecency, for the sake of the tenth part of a chance of catching a husband; and to simper and leer at the indecencies of a *Grande Duchesse de Gerolstein,* in order to escape the dreaded imputation of a deficiency in *chic.*—*Lancet.*

---

*Amputation under Chloral.*—M. Noir relates this case more as a warning than as an example for imitation, although he suggests that some of the symptoms which arose might have been due to the idiosyncrasy of the patient. The man, 64 years of age, was an inmate of the hospital at Brionde on account of osteosarcoma of the tibia, which caused him such excessive pain that he eagerly sought for amputation. With the view of allaying his suffering, four grammes of solid hydrate of chloral were given him, which at first induced great agitation and much nausea, then gave rise to such deep sleep and great subsequent relief, that it was determined to employ this substauce as an anæsthetic next day during the operation. Accordingly five grammes were given, and in about two hours so deep a sleep followed that he was carried to the table and amputation performed without his making a movement or uttering a cry. Replaced in bed, still asleep, his body was observed to be very cold, and his pulse, which had been 120, became filiform and uncountable. He continued in a state of alarming coma until eleven and a half hours after the chloral had been administered. He then woke up in violent delirium, with vomiting and pain in the stomach. These symptoms continued for nearly eight hours, when they disappeared, leaving the patient in the extremest prostration. He was conscious, but could scarcely move or speak, and was ignorant of all that had passed. After some hours and a good night, though without sleep, all bad effects passed off.—*Gaz. des Hospitaux.*

---

*Carbolic Gylcerine and Plaster.*—Dr. T. E. Jenkins, of Louisville, has writen the following note to Mr. Proctor, Editor of the *American Journal of Pharmacy.*

"During our sojourn in Europe in 1867, you may remember Dr. Lister of Glasgow, was experimenting with carbolic acid, with a view to its use as a surgical dressing. He found that one of the most convenient and efficient modes of applying this agent was to incorporate it with glazier's putty. This was a good idea, for the putty is plastic, handy, cheap not uncleanly, and adapts itself well to any irregular surface. This close contact is advantageous in two ways—it brings the medicament in intimate connection with the parts to be healed, and it serves the important purpose of excluding the atmospheric air, with its myriads of germs of organisms, thus preventing the action, whatever it may be, of this supposed prolific source of trouble—spores floating in the air—upon sores and exposed denuded surfaces, to say nothing of the influence of atmospheric oxygen upon part of low vitality, and struggling to resist surrounding destructive agents and processes (its 'levelling' propensity?).

"This putty, however is not the best vehicle for the purpose intended, for it dries pretty rapidly and 'sets;' it becomes rigid and finally hard, thus getting into a condition calculated to do much harm and occasion great discomfort. Having been applied to overcome this difficulty, glycerine instantly suggested itself to me, and I proposed to make a putty with this valuable body instead of linseed oil. The experience of a year and more has established the great value of the improvement. The formula I framed gives a preparation possessing the proper consistence, and one which maintains its properties unimpared, when kept in close jars for a long time.

I. *Carbolic Glycerine.* (T. E. J.)

℞ Carbolic Acid,.................. 1 part.
Glycerine,........................ 4 parts.

Mix.

II. *Carbolic Plaster.* .(T. E. J.)

℞ Carbolic Glycerine 34 pts. by weight.
Prepared Chalk... 94 pts.

Mix well by kneading, and enclose in closely stoppered jars.

"This preparation will, I think, be found to be all that is desired."

---

*Solutions of Protoxide of Nitrogen.*—M. STANISLAS LIMOUSIN has published a long paper on protoxide of nitrogen, which presents several points of interest for pharmaceutists at the present time, because this gas has been a good deal employed recently to produce anæsthesia, more especially for dental operations.

After referring to the general history of the gas, M. Limousin states that, in the course of some experiments in 1866, he was much struck by the great solubility of protoxide of nitrogen in water. Having one day a bottle containing equal volumes of the gas and cold water, he was surprised to find that upon violent agitation the stopper was forced down into the bottle with a detonation. Upon repeating his experiments, he found the water, (at about 4° or 5° C.) was capable of dissolving its own volume of the gas at the ordinary atmospheric pressure. This solution has a pleasant, slightly sweet taste, and is more agreeable to drink than pure water. It communicates this particular taste to wine and other liquids with which it is mixed. When the solution is affected under pressure, several volumes of gas are dissolved by the water, and this sweetness becomes more manifest. The author finds this solution of the gas to be perfectly innocuous; he has drunk it in doses of two bottles a day, sometimes pure, sometimes mixed with wine, and it has produced only a slight excitement and sensation of warmth to the head, somewhat similar to the effects of alcohol.

Dr. Demarquay has also studied upon himself the effects of this solution. He has taken it for several days, and he states that it produces upon the digestive functions a very marked stimulant and aperient action.

M. Limousin also directs attention to the solution of protoxide of nitrogen in ether. He finds that when ether is maintained at a temperature of—12° C. by a freezing mixture, it is capable of absorbing eight times its volume of nitrous oxide. Prepared under these conditions, the saturated ether acquires remarkable properties. It volatilizes with so much greater rapidity than pure ether, and produces thereby such a diminution of temperature that it would probably produce very energetic effects if applied to produce local anæsthesia. A mixture of strong alcohol and ether, saturated with protoxide of nitrogen, introduced upon cotton into a decayed tooth, produced momentarily an instantaneous disappearance of the pain. The vapor of this ethereal solution possesses a slightly sweet taste. Introduced into the lungs it produces a very agreeable peculiar sensation, and loses the sharp irritating taste of ether inhalations, which, with some persons, augments the nervous excitement.

M. Limousin also details a number of experiments intended to assist in forming an explanation of the physiological properties of nitrous oxide. He shows that this body is in fact a somewhat unstable compound, decomposed under comparatively feeble influences into oxygen and nitrogen; he regards it therefore as an oxidizing substance, and capable of acting as such when introduced into the animal economy. In the course of his paper the author mentions that bags or vessels of caoutchouc cannot be used for the preservation or transference of the gas, because this substance allows a very rapid diffusion of the gas to take place. *Druggists Circular.*

---

*Furrows on the Nails as the Result of Illness.*—In a recent number of the *Lancet*, Samuel Wilks, M.D., Physician to Guy's Hospital, mentions the fact that the traces of a past illness are indicated by markings or furrows on the nails. His own distinct knowledge of the fact that the nails become altered in disease was obtained, when a non-professional gentleman observed the circumstance for himself, and was so much interested in it that he referred the matter to a distinguished natural philosophor. It was after a severe attack of diarrhœa, which caused almost as much prostration as Asiatic cholera, that he discovered a white line or depression at the roots of the nails. Having formed a pretty accurate idea of their rate of growth, he was convinced that the markings corresponded with the date of illness. These marks are caused by a slight furrow, which is found more especially on the middle of the nail, and more distinct on that of the thumb. They point, no doubt, to a sudden arrest of the nutritive process during the time of the illness, and herein lies the interest of the observation. In cases of fever it is known that the most profound changes take place in all the tissues of the body. In scarlet fever, the whole of the epithelial surface within and without the body is affected, and, as a result, we may witness a desquamation of the cuticle, falling of the hair, and separation of the nails. When the fever is at its height, we can have then little doubt of the changes taking place in the tissues, and can feel no surprise that the nails show evidence of the former conflagration. As the patient recovers, and a new cuticle forms, and the hair begins to grow, the nail proceeds to shoot forward afresh, and it s not long before the latter exhibits a transverse furrow, indicative of the previous illness. It is possible, therefore to ascertain the date of the attack. Physiologists say that the thumb nail grows its whole length twice in a year, and thus it follows that if the furrow be found in the middle of the nail, the illness occured three months before. This fact may serve for a limited period, like "foot prints on the sands of time," as some additional proof of previous serious illness. For instance, a patient with a cardiac disorder stated that he had an illness three months before, and on his nails some transverse markings were found; also another suffering from phthisis said that his illness resulted from an inflamation of the lungs, occurring a few weeks previously, and on his nails also some distinct lines were discovered. That a severe diarrhœa could produce such a cessation of the nutritive process as to exhibit its effects on the nails, is a fact for which he was unprepared had it not been apparent to his eyes. In conclusion, he promises to present to his fellow-practitioners at some future date, further accurate clinical observations with reference to the subject.—*Medical Record.*

---

*Why do we Oil our Whetstones?*—GREAT men sometimes give utterance to arrant nonsense. Professor Tyndal, in his work, *Heat considered as a Mode af Motion*, asks the same question that we have placed as a caption to this note, and replies, in general terms, that it is to prevent friction. We have seen it stated somewhere that a little carbolic acid dissolved in water which is used to moisten a whetstone or grindstone, will greatly increase the amount of friction, and thus promote the action of the stone on the steel instrument. If this he true, and there be no unforseen drawback, carbolic acid will prove invaluable to all who have to sharpen tools or grind metallic surfaces. We oil our hones for several reasons. The first is, that almost all stones, unless oiled, become glazed or burnished on the surface, so that they no longer abrade the metal. The second reason is, that most stones, after being oiled, give a finer edge than they do in a dry or merely wet state. The pores of the stone become in a measure filled up, and while the action is rendered continuous, its character is altered. A dry stone is very apt to give a wire-edge to a tool, and although this sometimes happens when oil is used, yet it does not occur nearly so often. Some stones work better with water than with oil.—*Druggists Circular.*

*Skin Diseases.*—The following formula as an application in skin diseases, attended with little discharge, will be found satisfactory.

℞ Ferri pulv., ℥j.

Cinchonæ rubræ pulv., ℥ss.

Boracis pulv., ʒij.

Ol. Morrhuæ, q.s. for unguent. M.

A combination which forms a coating as impervious to air as collodion, and which the writer has employed with happy results in several cases of eczema, is prepared by adding ʒij. quiniæ sulph. to ℥ss. aa. tinct. ferri. chl. and tinct. cinchonæ, the parts to be painted with two or three coats.—*Med & Surg. Reporter.*

---

*To Prevent Tetanus*—To relieve from the terrible effects of running a nail in the foot of a man or horse, take peach leaves, bruise them, apply to the wound, confine with a bandage. They cure as if by magic. Renew the application twice a day if necessary, but one application usually does the work.—*Druggists Circular.*

---

*A New Cement.*—The "Journal de Chimie Medicale" states that an excellent cement may be made by dissolving 1 part of amber in 1½ part of sulphide of carbon. This liquid is applied by a brush to the surfaces it is wished to unite, and on pressing them together the cement dries almost immediately.—*Druggists Circular.*

---

